# The effectiveness of the community reinforcement approach (CRA) in the context of quality of life and happiness among people using drugs

**DOI:** 10.3389/fpubh.2024.1229262

**Published:** 2024-03-05

**Authors:** Muhammad Talha Khalid, Muhammad Tahir Khalily, Tamkeen Saleem, Fahimeh Saeed, Sheikh Shoib

**Affiliations:** ^1^Department of Psychology, International Islamic University, Islamabad, Pakistan; ^2^Faculty of Social Science & Humanities, Shifa Tameer-e-Millat University, Islamabad, Pakistan; ^3^Department of Neurosciences, School of Medical Sciences, Universiti Sains Malaysia, Penang, Malaysia; ^4^Department of Clinical Psychology, Shifa Tameer-e-Millat University, Islamabad, Pakistan; ^5^Psychosis Research Center, University of Social Welfare and Rehabilitation Sciences, Tehran, Iran; ^6^Department of Health, Kashmir, India

**Keywords:** quality of life, happiness of life, addiction, integrated approach, Community Reinforcement Approach

## Abstract

**Introduction:**

The Community Reinforcement Approach is an evidence-based treatment modality for alcohol and drug addiction treatment with proven efficacy and cost-effectiveness. The present study investigated the effectiveness of the Community Reinforcement Approach (CRA) in the context of quality of life among drug addicts.

**Materials and methods:**

A total of 60 inpatient substance abusers post detoxification in Fountain House, Lahore, Pakistan, participated in this study. Fountain House was selected as the Minnesota model is primarily used there. Therefore, a new treatment approach was introduced to investigate its effectiveness for individuals with substance abuse. A randomized 12-week trial was conducted as a substance use disorders (SUDs) treatment program. Persons with SUD (i.e., identified patients) enrolled in a residential treatment program were randomized into the integrated model of the Community Reinforcement Approach (CRA) and traditional Minnesota model treatment (*n* = 30), and traditional Minnesota model treatment only (TMM; *n* = 30). All the participants in the experimental group attended the group therapy sessions and other activities in the facility in addition to the treatment conditions. The participants attended the individual therapeutic sessions, which were conducted according to the CRA guidelines used in the experimental group. In this study, each individual in the CRA treatment group received 12 one-to-one sessions ranging from 45 min to 1 h. The WHOQOL-BREF scale and Happiness Scale (1) were used for data collection.

**Result:**

The results showed a significant increase in the quality of life of participants in the treatment group with CRA compared with the control group with TMM. The findings also indicated that the individuals in the treatment group with CRA had improved levels of happiness compared with individuals with TMM.

**Discussion:**

The CRA is an effective and adaptable treatment approach that works well in combination with other treatment approaches. The proven efficacy, compatibility, and cost-effectiveness distinguish it from other treatment methods.

**Implications:**

The CRA should be adapted, assessed, and evaluated further, especially in Pakistan, where there is a pressing need to adopt an effective treatment strategy for addiction problems.

## 1 Introduction

Drug abuse and addiction is a serious problem in Pakistan with numerous causal factors (2–4), including recreation, pleasure, social, medical, and psychological reasons (5). Currently, there are approximately seven million drug addicts, with an alarming number of injecting addicts, which is also a public health problem (6). It is not only a problem for the individual who uses or abuses drugs but also their loved ones (7).

For this reason, it is not a simple phenomenon and instead presents several challenges that require ameliorating multidimensional strategies (8). In addition, drug addiction increases the risk of numerous serious health issues, such as hepatitis, liver failure, heart attack and pulmonary arrest, HIV and AIDS, premature death, and bearing children with a range of mental and physical illnesses.

### 1.1 Historical perspectives and the disease model

Historical, cultural, and geographical values are very important in the onset and progression of drug abuse; however, sociodemographics, financial status, and psychological dynamics are key determinants of drug addiction. The results from a study conducted in Pakistan show that the different facets of an individual’s life are very much correlated with substance use, of which unemployment and post-traumatic stress disorder (PTSD) are two of the most crucial (9–11). Likewise, societal and environmental aspects, such as the availability and accessibility to drugs, increase the risk of and vulnerability to drug misuse.

Historically, the problem of addiction was thought of as an individual problem in the context of the disease model (12), and the main focus was placed on individual factors and ignored the environmental factors, which are equally important. Availability, accessibility, and acceptability play an important role in the spread of drug addiction.

The disease model states that addiction is a disease (12), which is irreversible. It can be treated only by lifelong sobriety or abstinence. It only considers individual factors and ignores the importance of the societal role in the initiation, sustenance, and relapse of addiction.

Therefore, the treatment approaches grounded in the disease model mainly focus on bringing about changes in the individual by improving their deficiencies while ignoring the environmental factors. Consequently, there has been a high level of relapse when using strategies that only focus on the deficiencies of individuals.

### 1.2 Community reinforcement approach

Keeping in view the importance of environmental factors, Hunt and Azrin (13) crafted the Community Reinforcement Approach (CRA) while striving to restructure a patient’s community so that a recovery was more rewarding than a life of addiction. The CRA was established on the principle that environmental settings and uncertainties are important for encouraging or discouraging substance use (14). The CRA rationales that an individual’s recovery from drugs is highly affected by their surrounding environment (15). The CRA uses familial, social, recreational, and vocational reinforcers to assist drug addicts in the recovery process. It is a comprehensive behavioral program for drug addiction treatment.

The CRA is a wide-ranging cognitive and behavioral approach for addictions and has been successfully used with inpatient (16) and outpatient drug addicts with high efficacy (17). Likewise, it has been studied with homeless individuals and resulted in good treatment outcomes (18).

Three meta-analytic studies cited the CRA as a highly cost-effective treatment program (19). In another evaluative study of the most economical treatment methods for drug addiction (alcoholism), the CRA was number one among 24 treatment methods (20). During the past 25 years, several studies have proven the effectiveness of the CRA in the treatment of substance use disorders.

Regarding the evaluation of the CRA itself, studies indicate its significant efficacy. Azrin initially assessed the program among alcohol-dependent inpatients in two studies (13, 16), revealing that the CRA outperformed the hospital’s Alcoholics Anonymous program in reducing drinking. Additionally, participants in the CRA program exhibited superior job and family relationship outcomes. Azrin later adjusted the program slightly for testing with outpatients at a rural alcohol treatment agency (21), once again demonstrating its superiority over the comparison condition.

However, a broader study in the 1990s showed mixed results, despite demonstrating immediate benefits from the CRA (22). Nonetheless, various comprehensive reviews, meta-analyses, and randomized controlled trials affirm the CRA’s high effectiveness compared with other treatments for alcohol, cocaine, and opioid use (22–27).

A recent short-term test of IA-CRA in Pakistan demonstrates its ability to help individuals achieve abstinence from cannabis use and provides moderate relief for depressive and anxiety symptoms. These positive effects persist for a minimum of 36 weeks after the intervention (28).

### 1.3 Study significance

In practice, individuals with drug addiction problems have diverse needs, and there is no single treatment modality that can meet all of these needs or is effective for all types of drug addiction. Moreover, traditional methods of drug addiction treatment are not sufficiently effective and lack research, which is also one of the main reasons for the high relapse ratio.

In Pakistan, there is lack of studies on the CRA with substance users in this context. This research is innovative as, through empirical study, it assesses the effectiveness of an integrative strategy including the CRA and Minnesota model, which form a unique combination despite their theoretical and philosophical differences. Therefore, this study aims to test the effectiveness of this integrative model in a Pakistani population, in which addiction is a major and serious problem and requires a treatment of proven efficacy.

There is a dire need to integrate traditional treatment modalities with effective and evidence-based approaches, such as the CRA, to meet the present demand and challenges of our society. The CRA has shown high efficacy in multiple clinical trials and also when it has been integrated with other treatment methods, such as pharmacological support, contingency management (CM), motivational interviewing (MI), and family therapy (29). The compatible integration of different approaches can provide the synergized effect with highly effective outcomes. The CRA has not been combined in such a way in which its core strengths and those of another treatment have been assessed and evaluated.

### 1.4 Theoretical framework: the CRA, quality of life, and happiness

**Figure 1:**
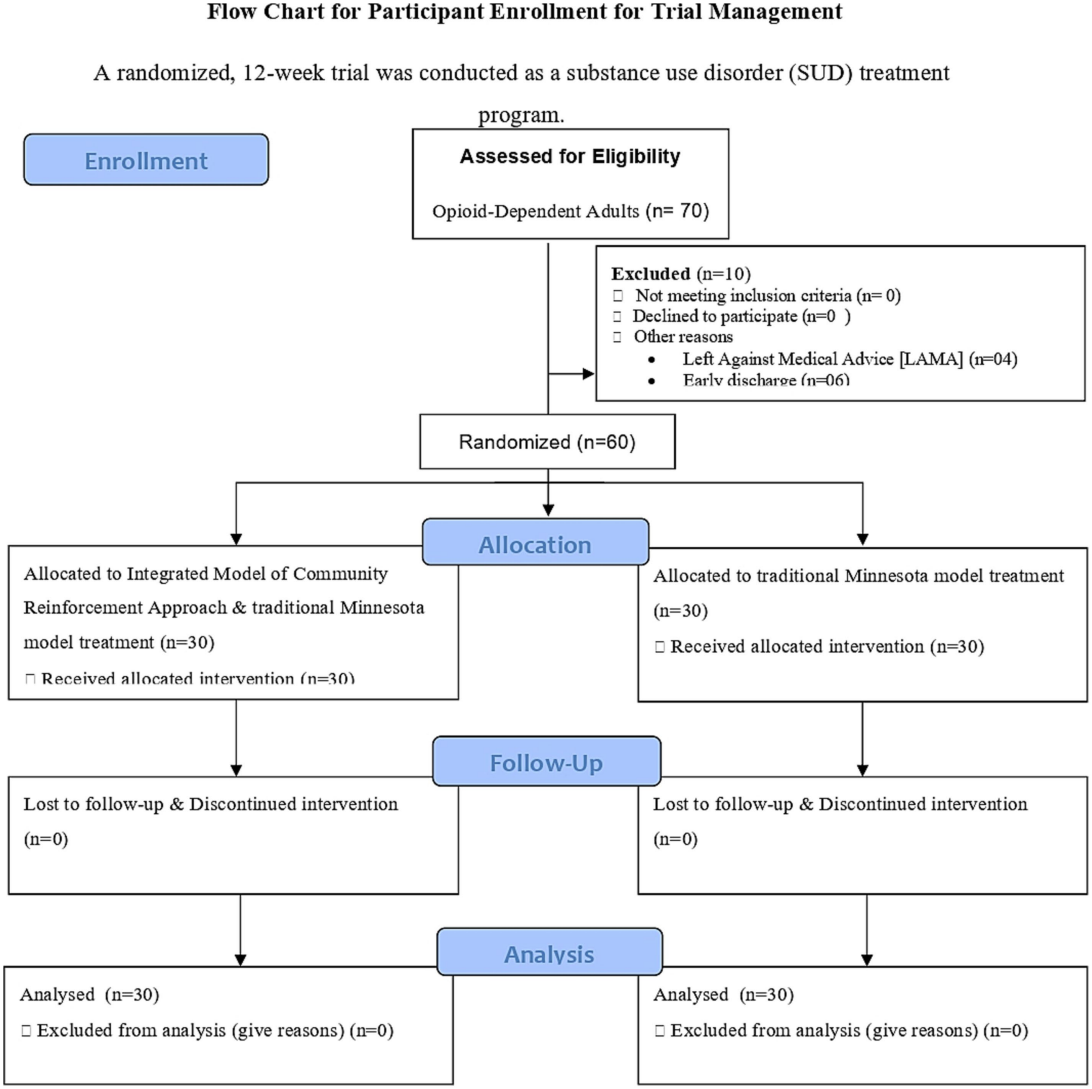
Flow chart of participant enrollment for trial management.

The CRA serves as the central therapeutic model, focusing on behavioral strategies to address substance use disorders. It emphasizes positive reinforcement for non-substance-related behaviors, improving the social, vocational, and familial aspects of the lives of individuals (30) (Figure 1).

The research and development of the Community Reinforcement Approach (CRA) in addressing substance use disorders are influenced by several theoretical perspectives:

Behavioral Theory: Operant Conditioning: The CRA draws heavily from operant conditioning theory, particularly B.F. Skinner’s principles, emphasizing how behaviors are reinforced or extinguished based on their consequences. The CRA employs positive reinforcement to promote positive behaviors that are incompatible with substance use.

Social Learning Theory: Bandura’s social learning theory underscores the impact of social environments on behavior. The CRA acknowledges the role of social and environmental factors in maintaining or changing substance use behavior, focusing on modifying the individual’s surroundings to support sobriety.

Motivational Enhancement Theory: The CRA incorporates motivational enhancement principles to increase an individual’s motivation for change. It aims to increase intrinsic motivation by helping individuals recognize and reinforce positive changes in their lives.

Social Reinforcement Theory: The CRA leverages positive reinforcement theory by fostering positive social interactions and reinforcing non-substance-related behaviors. It aims to create a social context that rewards sober behaviors.

Health Behavior Change Models: Elements from models such as the Transtheoretical Model (Stages of Change) inform the CRA by recognizing different stages of readiness for change in individuals and tailoring interventions accordingly.

Ecological Systems Theory: Environment and Context: The CRA considers individuals within their broader ecological context, acknowledging the influence of various systems (family, work, and community) on substance use behaviors.

The CRA framework, when applied effectively, contributes to improvements in various aspects of the lives of individuals, ultimately enhancing their QoL and fostering happiness. By targeting behaviors and environments that contribute to well-being, CRA interventions can create a positive ripple effect on overall life satisfaction and happiness.

This theoretical framework aligns CRA interventions with broader dimensions of QoL and happiness, emphasizing the holistic nature of behavioral interventions in promoting well-being beyond solely addressing substance use disorders. The strength of the CRA lies in its tailored approach, addressing each individual’s unique circumstances and reinforcing behaviors that lead to a substance-free lifestyle.

## 2 Materials and methods

### 2.1 Purpose of the study

The aim of this study was to evaluate the effectiveness of this integrated model within the Pakistani population, addressing the significant and pressing issue of addiction that demands treatment with established efficacy.

### 2.2 Participants

The sample consisted of 60 participants with substance use disorders in Fountain House, Lahore, Pakistan, between October, 2016, to February, 2017. The 30 participants were randomly allocated to each group, i.e., the experimental (CRA) and control (TMM) groups. The experimental group received a 12-week intervention based on an integrated model of the Community Reinforcement Approach (CRA) and traditional Minnesota model treatment, and the control group received the treatment via the traditional Minnesota model (TMM) only at the treatment facility. The adult inpatients with a chemical addiction to cannabis, heroin, and alcohol were included in the study. The age range was 15 to 50 years. The consent for inclusion in the study was taken from the participants; in scenarios in which participants were unable to provide consent, their parents/guardians/attendants completed the consent form. The participants were not diagnosed with any other major psychological or psychiatric issues. The participants who were admitted for non-chemical addiction or with comorbidity, as well as outpatients, were excluded from the study.

### 2.3 Procedure

The integration of the Community Reinforcement Approach (CRA; Table 1) and traditional Minnesota model was applied to the experimental group, whereas the control group only received the traditional Minnesota model. All participants were still attending group therapy sessions and other activities in the facility.

**Table 1:** The community reinforcement approach protocol.

Session 1	Treatment orientation, rapport building, functional analysis for using behavior, functional analysis for pro-social behavior	Psycho-education. Identification of external and internal triggers, drug use behavior, short-term positive and long-term negative consequences of drugs. Identification of the triggers and consequences of non-using activities. Assessment of happiness with life and quality of life scales.
Session 2	Happiness Scale, Quality of Life Scale, CRA Treatment Plan	Rating of happiness in 10 different areas of life in the present day. Assessment of present quality of life. Discuss and plan the structured goals of counseling in line with 10 areas of life rated on the happiness scale.
Session 3–4	Behavior skills	Teaching of communication and problem-solving skills using CRA guidelines and communication and Problem-solving worksheets
Session 5–6	Job skills	Assessment of occupational skills and guidance for searching for a suitable job, role plays for job interviews and reflections.
Session 7	Social skills	Identification of new replacement social and recreational activities. Selecting highly reinforcing alternative ways to satisfy their recreational needs.
Session 8–9	Relapse prevention	Working on internal and external triggers of drug use. Teaching drug refusal skills and self-monitoring for early signs of probable relapse.
Session 10–11	Relationship counseling for married participants, relationship happiness scale, perfect relationship worksheet, self-reminder to be nice	Rating of relationship happiness in 10 areas of life. Goal setting for relationships by use of perfect relationship worksheet. Practice communication skills, role plays with spouse, introduction and use of daily reminders to be nice to each other.
Session 12	Termination	Termination of counseling sessions, post-test measures taken. Reminders for managing relapse and relationship provided.

After initial working (permissions, sample selection, sampling, and allocation of groups), the duration of treatment application was 3 months. The researchers followed the “Clinical Guide to Alcohol Treatment” (1) as a treatment protocol. At the end of the treatment, post-tests were conducted for all of the individuals who took part in the study. A comparison was made to check the significant differences between the applied treatments.

### 2.4 Measures

#### 2.4.1 The WHOQOL-BREF scale

The brief version of quality of life consisted of 26 items. It is a five-point Likert scale. This scale expresses quality of life in four domains of an individual: physical health, psychological health, social relationship, and environment (31). The WHOQOL brief scale is a detailed, cross-cultural, valid, and reliable scale (α = 0.90) for evaluating the quality of life of an individual (32).

#### 2.4.2 Happiness scale

This scale is used in the Community Reinforcement Approach (CRA) to evaluate the current happiness with life of an individual in 10 different areas of life. It is a 10-point Likert scale. The therapist/counselor uses the responses to design counseling goals. It is a self-administered scale in which the respondent asks himself “How happy am I with this area of my life?”; they rate each area from 1 to 10 with regard to how they presently feel. It is a reliable (α = 0.82) and valid scale (33–35).

## 3 Data analyses

Descriptive analysis was used for the sociodemographic and clinical characteristics of the sample. A t-test for the independent sample was used to analyze the data.

## 4 Results

### 4.1 Participants characteristics

The clinical characteristics of the participants showed that the mean age of initiating smoking was 15 years, ranging from 5 to 36 years. The mean age of initiating drug use was 18 years, ranging from 9 to 41 years. Of the participants, 18.3% had drug users in their family, whereas 81.7% did not have a drug user in their family. Most participants were heroin users (68.3%); among others, 28.3% were cannabis users and 3.3% were alcohol users. The highest route of drug administration was smoking (40%), followed by the inhalation of fumes (23.3%). The percentage of snorting and IV injection was the same (16.7%). Swallowing had the lowest percentage (3.3%). The median number of treatments was 1, with a range of 1 to 16. Of the participants, 58.3% were going through treatment for the first time (Table 2).

**Table 2:** Clinical characteristics of the participants (*n* = 60).

Variables	Categories	N	%	M	SD	Range
Age started smoking		60	100	15	5.5	5–36
Age started using drugs		60	100	18	6.7	9–41
Drug user in family						
	Yes	11	18.3			
	No	49	81.7			
Drug of choice						
	Cannabis	17	28.3			
	Heroin	41	68.3			
	Alcohol	2	3.3			
Route of administration						
	Smoking	24	40			
	Snorting	10	16.7			
	Inhaling fumes	14	23.3			
	IV Injection	10	16.7			
	IM Injection	0	0			
	Swallowing	2	3.3			
No. of treatments						1–16
	1st	23	53.3			
	2^nd^	8	13.3			
	3^rd^	7	11.7			
	4^th^	3	5.0			
	5^th^	2	3.3			
	5+	8	13.4			
No. of relapses				1	3.12	0–15
	Never	32	53.3			
	1st	8	13.3			
	2^nd^	7	11.7			
	3^rd^	3	5.0			
	4^th^	2	3.3			
	5^th^	1	1.7			
	5+	7	11.7			

### 4.2 Psychometrics of the study variables

Table 3 shows the reliability of the Happiness Scale, which was 0.74. The Cronbach alpha reliability of the QOL scale was 0.83 for the present study.

**Table 3:** Psychometric properties of the major variables in the study (*n* = 60).

			Range					
Measurements	items	α	Min	Max	M	SD	Skew.	Kurt.
QOL	26	0.87	100	379.17	278.72	55.92	−0.34	0.40
PHYS	7	0.70	35.71	100	77.02	13.84	−0.30	0.08
PSYCH	6	0.53	37.5	100	70.9	14.19	−0.37	−0.37
SOCIAL	3	0.39	25	100	65.25	18.77	−0.07	−0.59
ENVIR	8	0.74	34.38	100	68.96	17.05	−0.17	−0.94
HS	10	0.74	34	93	73.9	12.77	−1.06	1.41

QOL, Quality of Life Scale; PHSY, physical domain; PSYCH, psychological domain; SOCIAL, social relationship domain; ENVIR, environment domain; HS, Happiness Scale.

### 4.3 Treatment analysis

Table 2 provides a comprehensive overview of the clinical characteristics related to substance use among the 60 participants in the study. It includes details such as age of initiation of smoking and drug use, family history, drug preferences, methods of drug intake, treatment frequency, and relapse instances, which are essential factors in understanding and analyzing substance use behaviors and treatment outcomes.

The ages at which participants started smoking ranged from 5 to 36 years old, indicating a wide range of initiation ages within the group. On average, participants began smoking at around 15 years old (standard deviation [SD] = 5.5: there was a variability of approximately 5.5 years around the mean age of initiating smoking among the participants). The ages at which participants started using drugs ranged from 9 to 41 years old, demonstrating that there was a wide range of initiation ages in this group. On average, participants initiated drug use at around 18 years old (M = 18, SD = 6.7), with a variability of approximately 6.7 years around the mean age of initiating drug use among the participants. Eleven participants (18.3%) had drug users in their family, whereas 49 participants (81.7%) did not. This indicates a minority of participants had a family history of drug use. Additionally, Table 2 shows the drug of choice among participants. Cannabis was the choice for 17 participants (28.3%), heroin for 41 participants (68.3%), and alcohol for 2 participants (3.3%). Heroin stands out as the most prevalent choice among the participants. The most common routes of drug administration were smoking (40%), inhaling fumes (23.3%), and snorting (16.7%). IV injection and swallowing were less common methods. Participants received between 1 and 16 treatments, with varying frequencies for different treatment cycles. This shows a diversity in the number of treatments each participant underwent.

Similarly, participants also experienced relapses ranging from 0 to 15 instances, with different frequencies for each. This suggests a wide variation in the number of relapses among participants, with some experiencing multiple relapses.

Table 3 presents the psychometric properties of various major variables measured via study instruments in the investigation, providing information on the reliability and characteristics of these measurements. The “Quality of Life” measure, comprising 26 items, demonstrated notably high internal consistency (α = 0.87), with scores averaging 278.72 and displaying a moderately peaked distribution. “Physical Health” and “Environmental Health” exhibited acceptable internal consistency (α = 0.70 and 0.74, respectively), with the former showing mildly negatively skewed scores and the latter demonstrating nearly symmetric scores. Conversely, “Psychological Health” and “Social” aspects displayed relatively lower internal consistency (α = 0.53 and 0.39, respectively), signifying more variability within their item set, while “Happiness,” measured with 10 items, presented good internal consistency (α = 0.74) despite a moderately negatively skewed distribution and notably heavy-tailed scores. These findings collectively highlight varying degrees of reliability and distributional characteristics across distinct domains, suggesting the nuanced nature of quality of life and happiness among the study’s participants.

An independent samples t-test in Table 4 showed that QOL scores were significantly higher for the experimental group (M = 299.06, SD = 60.04) than for the control group (M = 258.38, SD = 43.61, t (58) = 3.00, *p* < 0.01). The effect size of 0.7 indicates a moderate effect size; there is a noticeable and meaningful difference between the two groups, indicating that the CRA is an effective approach for drug abuse treatment as it increases the overall quality of life of individuals. The scores for the physical domain were significantly higher for the experimental group (M = 83.12, SD = 12.55) than for the control group (M = 70.85, SD = 12.50, t (58) = 3.75, *p* < 0.001). The scores for the psychological domain were significantly higher for the experimental group (M = 78.02, SD = 10.62) than for the control group (M = 64.03, SD = 13.95, t (58) = 4.32, *p* < 0.001). The scores for the social relationship domain were significantly higher for the experimental group (M = 71.26, SD = 17.99) than for the control group (M = 59.44, SD = 18.01, t (58) = 2.53, *p* < 0.05). The scores for the environmental domain were significantly higher for the experimental group (M = 74.14, SD = 18.03) than for the control group (M = 63.96, SD = 14.67, t (58) = 2.38, *p* < 0.05). The effect size for four domains varied from a moderate to a large effect; 1.13 is the highest effect size for the psychological domain, which suggests a substantial and noteworthy difference between the groups being compared. The scores for the Happiness with Life Scale were significantly higher for the experimental group (M = 79.43, SD = 8.68) than for the control group (M = 68.47, SD = 13.90, t (58) = 3.70, *p* < 0.001). An effect size of 0.95 for happiness between an experimental group and a control group indicates a substantial and noteworthy difference in terms of happiness levels between the two groups.

**Table 4:** A t-test analysis of the experimental and control groups with regard to the variables of quality of life and its subdomains and happiness with life (*n* = 60).

		Experimental group1 (*n* = 30)		Control group1 (*n* = 30)				95% CI		
	Variable	*M*	*SD*	*M*	*SD*	***t(58)***	*p*	LL	UL	Cohen’s *d*
1	QOL	299.06	60.04	258.38	43.61	3.00	0.004	13.56	67.80	0.78
2	PHYS	83.12	12.55	70.85	12.50	3.75	0.000	5.67	18.62	0.98
3	PSYCH	78.02	10.62	64.03	13.95	4.32	0.000	7.52	20.47	1.13
4	SOCIAL	71.26	17.99	59.44	18.01	2.53	0.014	2.46	21.18	0.66
5	ENVIR	74.14	18.03	63.96	14.67	2.38	0.021	1.62	18.74	0.62
6	HS	79.43	8.68	68.47	13.90	3.70	0.000	5.18	17.16	0.95

A t-test analysis between the experimental and control groups with regard to the variables of quality of life and its subdomains and happiness with life (*n* = 60).QOL, quality of life; PHYS, physical domain; PSYCH, psychological domain; SOCIAL, social relationship; ENVIR, environmental domain; HS, Happiness Scale; M, mean; SD, standard deviation; LL, lower limit; UL, upper limit; Cl, confidence interval.

## 5 Discussion

The primary aim of this study was to evaluate the effectiveness of the community reinforcement approach on the quality of life of drug addicts when it is integrated with the traditional Minnesota treatment method and compared with individuals who received traditional treatment only.

The Community Reinforcement Approach stands as an evidence-based treatment method for addressing substance-abuse disorders and is known for its proven effectiveness and cost efficiency. The present study specifically delved into the impact of the Community Reinforcement Approach (CRA) on the Quality of Life of individuals struggling with drug addiction. The findings highlight a notable improvement in the quality of life among participants in the treatment group utilizing the CRA compared with the control group undergoing TMM. Moreover, levels of happiness were significantly higher in the treatment group employing the CRA than in the control group with TMM. The CRA proved itself as an effective and adaptable treatment strategy, showcasing its ability to synergize effectively with other treatment approaches.

Other studies have confirmed that the Community Reinforcement Approach is an extensive behavioral treatment strategy designed to address challenges related to substance use, emphasizing the management of its effects on social, occupational, and vocational aspects (13, 15, 22, 36). When contrasted with a placebo, the CRA shows a favorable effect on fostering abstinence while also exerting a positive influence on depressive and anxiety symptoms, along with improving overall quality of life (28).

The present study also explored the sociodemographics of the study participants. In Pakistan, the continuously increasing population causes various issues, including drug addiction. When a population increases, the diffusion of drug abuse also increases (an analysis of drug abuse networking in Pakistan). The results show that the number of siblings (81.7% have four or more) and birth order (23.3% are first born and 21.7% are middle born) may be important factors for the initiation of drug addiction. A previous study by Argys et al. (37) obtained similar results and found that the middle-born and last-born individuals are more likely to use drugs and be sexually active than their first-born siblings. The outcomes can vary culturally but these factors are important and need further investigation.

In the present study, the mean onset age of drug use was 18, which indicates the desperate need for drug education and prevention programs for adolescents and even younger people so that they can avoid addiction while living in an environment full of drug addiction cues. A previous study by Lloyd et al. (38) showed that drug use in early age, such as the use of tobacco, correlates with later drug misuse. The results of a study conducted by (39) suggested the use of goal settings for harm reduction and classroom approaches in school drug education.

There are so many treatment modalities for the problem of addiction, but each model has its own strengths and limitations. No single treatment modality has been adopted as a universal treatment model for the treatment of addiction. Moreover, there is no uniform treatment model that is appropriate for every individual with a drug abuse problem. The National Institute on Drug Abuse (NIDA) suggested that tailor-made treatment plans and interventions are critical for the rehabilitation of drug addicts so that they can become a positive part of society. Integrated approaches can result in more effective outcomes than a single or tunnel-viewed model.

### 5.1 Limitations and recommendations

The research had some limitations because of the small sample size and treatment engagement problems due to LAMA (left against medical advice), family dissatisfaction with the treatment, economic reasons, the resistance of staff, and the administration at the treatment center. The authors tried to reduce limitations by continuously assessing and updating treatment manuals or guidelines based on feedback, new research findings, or clinical experiences, which can increase the CRA’s relevance and effectiveness.

The inability to enroll the control group participants in the Community Reinforcement Approach (CRA) treatment due to time constraints and consent limitations represents a significant limitation of the study. This limitation may have impacted the ability to directly compare the effectiveness of the CRA with the traditional Minnesota Model treatment within the same study sample. Future research endeavors aiming to compare different treatment modalities should consider strategies to overcome such limitations, such as ensuring a sufficient time for enrollment, obtaining comprehensive consent protocols, or implementing alternative study designs that account for these challenges without compromising the study’s integrity.

Despite all these issues, there is a dire need to work on the CRA and its integration approach with present treatment methods. This is the only way to provide quality treatment for drug addiction. It may be adapted, assessed, and evaluated further in this regard, especially in Pakistan, where there is a pressing need to adopt and adapt treatment strategies for addiction problems with proven efficacy. This integrated method can bring about a revolution in the field of drug addiction treatment.

Future researchers delving into similar topics could benefit significantly from the integration of innovative qualitative methodologies such as Online Interpretative Phenomenological Analysis (OIPA) and Community-Based Participatory Research (CBPR). Integrating OPV and OIPA from a CBPR perspective can further explore and increase the impact of CRA-based interventions on individuals with SUD. This approach would capture rich lived experiences, potentially leading to improvements in CRAs tailored to the needs of the affected communities.

The integration of these methodologies aims to authentically capture the intricate nuances of thoughts, emotions, mental images, and behaviors stemming from individuals’ unique lived experiences. By drawing directly from these lived experiences, researchers can establish a robust foundation for understanding and addressing the topic effectively.

Moreover, the inclusion of Online Photovoice (OPV) as another innovative qualitative method could further enrich research endeavors. OPV’s capacity to allow participants to express their experiences with minimal manipulation provides an avenue to glean genuine and unfiltered insights into how the CRA functions in the context of substance use disorders.

## 6 Conclusion

This study aimed to assess the usefulness of the Community Reinforcement Approach in the context of quality of life when it is combined effectively with the traditional treatment method. The results show a notable increase in the quality of life of individuals receiving the combined treatment compared with those who receive the traditional treatment. Likewise, the happiness of the participants with the integrated method also increased compared with the participants in the Minnesota model treatment.

The integration approach can achieve much better results than any single treatment modality. We can integrate, customize, and tailor the strengths of different modalities with respect to the culture, type of addiction, and treatment method (indoor and outdoor). The CRA is highly flexible and compatible and is proven to be effective in combination with different treatment methods for substance use disorders.

### 6.1 Utilization of research results

This study can be very helpful in the planning and policymaking for the treatment of substance use disorders. In Pakistan, the traditional methods do not support current needs. Incorporating the CRA into traditional treatment methods will achieve great results in the treatment of drug addiction because it is a flexible, compatible, and highly effective treatment approach with proven efficacy. The best thing is that we can implement it according to cultural norms and traditions. It is the right time to introduce CRA in our country. This study offers a great initiative, to use this integrative model of the Community Reinforcement Approach and Minnesota Model for the treatment of addiction as evidence-based strategy.

### 6.2 Implications

The present study’s implications extend across various domains:

- ***Mental Health:***

*Treatment Enhancement:* CRA’s effectiveness in improving quality of life and life satisfaction for individuals struggling with drug addiction can inform mental health professionals and clinicians about an evidence-based approach that goes beyond substance use treatment.

*Management in Functional Domains:* The positive impact of CRA on quality of life suggests its potential in managing co-occurring mental health issues in various domains of functioning, highlighting its relevance in integrated care models.

- ***Education:***

*Training and Curriculum:* Insights from this study could influence educational programs for mental health professionals, potentially incorporating CRA techniques into training curricula for addiction treatment.

- ***Research:***

*Further Investigations:* Encourages more research into CRA’s mechanisms, its adaptability to diverse populations, and its comparative efficacy with other treatment modalities, contributing to a more nuanced understanding of addiction treatment.

- ***Administrators:***

*Resource Allocation*: Administrators in healthcare settings might consider the integration of the CRA into existing treatment programs based on its proven effectiveness, potentially improving outcomes for individuals struggling with addiction.

- ***Services:***

*Treatment Planning*: The adaptability and compatibility of the CRA with other approaches could prompt service providers to consider its integration into existing treatment services, possibly enhancing their effectiveness.

This study’s findings suggest that CRA holds promise not only as a standalone treatment but also as a complementary approach in diverse settings, indicating its potential for broader implementation across mental health, education, research, administrative, and service domains.

## Data availability statement

The raw data supporting the conclusions of this article will be made available by the authors, without undue reservation.

## Ethics statement

The studies involving humans were approved by Department of Psychology Ethic review Committee. The studies were conducted in accordance with the local legislation and institutional requirements. Written informed consent for participation in this study was provided by the participants' legal guardians/next of kin.

## Author contributions

MLK and MHK: conception and design of the work, acquisition, analysis, and interpretation of data, drafting the manuscript and revising it critically for important intellectual content for final approval, and accountable for the integrity of the intellectual content. TS: the acquisition, analysis, and interpretation of data for the work, revising the manuscript critically for important intellectual content for final approval, and accountable for the integrity of the intellectual content. FS and SS revising the manuscript critically for important intellectual content for final approval, and accountable for the integrity of the intellectual content. All authors contributed to the article and approved the submitted version.
